# Impact of the COVID-19 Pandemic on Intestinal Parasitic Infection Positivity Rates Among Clinically Suspected Patients at a Tertiary Centre in Northern India: A Four-Year Retrospective Analysis

**DOI:** 10.7759/cureus.117132

**Published:** 2026-09-29

**Authors:** Pratiksha Kamboj, Deepika Chakraborty, Yogendra P Mathuria, Sandipta Charkraborty, Binal Mangroliya, Sindura Sudhakar, Tanisha Sharma, NIdhi Soni

**Affiliations:** 1 Microbiology, Government Medical College, Haridwar, Haridwar, IND; 2 Microbiology, Govind Ballabh Pant Hospital, New Delhi, IND; 3 Microbiology, All India Institute of Medical Sciences, Rishikesh, Rishikesh, IND; 4 Public Health, International Institute of Health Management Research, Delhi, IND

**Keywords:** covid-19, epidemiology, intestinal parasite, ivermectin, prevalence

## Abstract

Background: The COVID-19 pandemic changed healthcare-seeking behaviour and clinical priorities, potentially influencing the detection of intestinal parasitic infections (IPIs). Data evaluating these effects remain limited.

Objective: The primary objective was to assess changes in the laboratory positivity rate of intestinal parasitic infections (IPIs) among clinically suspected patients during the COVID-19 pandemic, including the period of empirical ivermectin use. The secondary objective was to compare IPI positivity rates across different age and gender groups over the study period.

Methodology: This was a retrospective laboratory-based observational study conducted at AIIMS Rishikesh from January 1, 2019, to December 31, 2022. Stool samples underwent macroscopic examination, saline and iodine wet-mount microscopy, and modified Ziehl-Neelsen staining for coccidian parasites. The timeline was divided into three policy-defined periods: pre-COVID (January 1 to December 31, 2019), early COVID-19/ivermectin-included period (January 1, 2020, to August 31, 2021), and late COVID-19/post-ivermectin period (September 1, 2021-December 31, 2022). We applied a chi-square test and Bonferroni correction. Effect sizes are reported as prevalence ratios (PRs) with 95% confidence intervals (CIs).

Results: Of 5,389 samples, 97 (1.80%) were confirmed positive for IPIs; 103 parasites were isolated from 97 patients, with five patients harbouring co-infections. Overall positivity rates were highest in the age group of 41-50 years (3.00%; 13/433) and comparable between male (1.88%; 64/3,405) and female (1.66%; 33/1,984) patients. The pre-COVID positivity rate (2.78%; 37/1,330) exceeded that of the early COVID period (1.14%; 17/1,493; PR = 2.44, 95% CI: 1.38-4.32; p = 0.001). Protozoa predominated, with Giardia lamblia being the most frequent (44.66%; 46/103 parasites isolated). Following ivermectin withdrawal, positivity declined transiently (0.14%; 1/692; 95% CI: 0.03%-0.81%) before resurging (2.24%; 42/1,874).

Conclusion: Laboratory positivity for IPIs among patients with clinical suspicion fell during the initial COVID-19 period, most likely due to decreased healthcare-seeking and changes in referral patterns rather than a real shift in parasite prevalence at the community level. The observed temporal association with ivermectin use is only hypothesis-generating because individual-level treatment information is not available. Prospective, controlled studies that confirm ivermectin exposure data are required.

## Introduction

Intestinal parasitic infections (IPIs) are a major threat to global health and affect millions of people worldwide [1]. These infections are caused by diverse parasites, including nematodes, trematodes, cestodes, and protozoa, which are widely distributed across tropical and subtropical regions [2]. Intestinal parasitic infections remain a major public health problem worldwide, affecting approximately 1.5 billion people, including 267 million preschool children and 568 million school-aged children, particularly in low- and middle-income countries. Children bear a disproportionate burden of infection because of increased exposure to contaminated environments and inadequate sanitation [3].

COVID-19 emerged as a rapidly growing pandemic in December 2019 following the detection of the first case in Wuhan, Hubei Province, China [4]. The causative agent, severe acute respiratory syndrome coronavirus 2 (SARS-CoV-2), has been responsible for over 6 million deaths, making it the most serious global health calamity since the 1918 influenza pandemic. Given the limited therapeutic options available at the outset, several repurposed drugs were explored to manage this novel infection, including ivermectin, an established antiparasitic agent used off-label based on proposed mechanisms of viral replication inhibition [5,6].

Along with its direct health effects, the COVID-19 pandemic had many substantial indirect impacts on healthcare systems and disease surveillance worldwide. Public health measures such as lockdowns, travel restrictions, and social distancing may have influenced the transmission of other infectious diseases, including IPIs. Changes in behaviour, such as improved hand hygiene, mask use, and reduced outdoor activities, may also have affected exposure to parasitic infections. Concurrently, the redirection of healthcare resources and research efforts toward COVID-19 resulted in reduced surveillance of parasitic diseases and limited data on their trends during the pandemic period.

This study was conducted at a tertiary care hospital in northern India to assess changes in the positivity rate of IPIs before and during the COVID-19 pandemic and to examine their distribution across different age groups and sexes.

## Materials and methods

A retrospective laboratory-based observational study was conducted in the Parasitology Section of the Department of Microbiology, All India Institute of Medical Sciences (AIIMS), Rishikesh. All stool samples received from January 2019 to December 2022 were included in this study, comprising 5,389 samples from patients of all age groups and both sexes across outpatient and inpatient departments.

Stool samples were received at the laboratory from patients referred for parasitological examination on clinician recommendation, predominantly in the context of gastrointestinal symptoms such as diarrhoea, abdominal pain, dysentery, bloating, nausea, vomiting, unexplained weight loss, or persistent gastrointestinal complaints, and in selected cases for indications including eosinophilia, immunocompromised status, or clinical suspicion of parasitic infestation. No standardised written symptom-based referral criteria were in place, and sample submission reflected routine clinical practice at this centre.

Stool samples were collected and subjected to standard stool investigations, including macroscopic examination for the presence of adult worms or their body segments and microscopic examination using saline and iodine wet mounts for protozoal cysts, trophozoites, and helminth ova and larvae. Modified Ziehl-Neelsen (mZN) staining was performed on a reflexive basis either when wet mount microscopy revealed morphological features suggestive of coccidian parasites or on the clinician’s recommendation. Acid-fast oocysts were identified based on standard morphological criteria and size, thus enabling detection of *Cryptosporidium *spp., *Cyclospora cayetanensis*, and *Cystoisospora belli*. As molecular or antigen-based assays were not routinely available, differentiation between morphologically identical species within the *Entamoeba histolytica/dispar/moshkovskii/bangladeshii *complex was not possible. Therefore, reported cases were interpreted as the *E. histolytica/dispar* complex, in accordance with routine diagnostic practice in resource-limited settings.

Duplicate samples from the same patient, samples with incomplete documentation, and leaking samples were excluded from the study. Laboratory-confirmed cases were considered positive, and the proportion of positive cases among suspected patients was considered the positivity rate. The study timeline was divided into three periods: (i) pre-COVID (January 1 to December 31, 2019, before inclusion of ivermectin in the treatment protocol), (ii) the COVID-19 period during which ivermectin was included in the treatment protocol (January 1, 2020, to August 31, 2021), and (iii) the subsequent COVID-19 period following its removal from the treatment protocol (September 1, 2021, to December 31, 2022). Individual-level ivermectin exposure could not be ascertained from the retrospective laboratory records; therefore, these periods represent policy-defined periods rather than confirmed individual-level ivermectin exposure [7].

Categorical variables are presented as frequencies and percentages. The overall positivity rate across the study timeline was compared using the chi-square test. Pairwise comparisons between individual time periods were performed using the chi-square test with Bonferroni adjustment. A p-value of <0.05 was considered statistically significant for the overall three-group comparison. For the three post hoc pairwise comparisons, the Bonferroni-adjusted significance threshold was set at α = 0.017 (0.05/3). Prevalence ratios (PRs) and odds ratios (ORs) with 95% confidence intervals (CIs) were calculated as effect size measures for each pairwise comparison. CIs for individual positivity rates were calculated using the Wilson exact method. Data analysis was performed using Stata 17.

## Results

A total of 5,389 patients with clinically suspected intestinal parasitic infections were included in the study. The maximum number of samples were received from the 21-30-year age group (n = 1,907; 35.38%), followed by the 31-40-year age group (n = 1,324; 24.57%) (Table 1). Among the total samples received, males constituted 63.18% (3,405/5,389), while females comprised 36.82% (1,984/5,389).

Of the 5,389 suspected cases, 97 (1.80%) were confirmed positive for intestinal parasitic infections. Within-group positivity rates varied across age groups, with the highest positivity rate observed in the 41-50-year age group (3.00%; 13/433), followed by the 11-20-year age group (2.56%; 8/312) and the 0-10-year age group (2.40%; 15/626). The 21-30-year age group, despite contributing the largest absolute number of positive cases (n = 23), had a within-group positivity rate of 1.21% (23/1,907), reflecting its disproportionately large representation in the tested population. Within-group positivity rates were comparable between males (1.88%; 64/3,405) and females (1.66%; 33/1,984), with no statistically significant difference between the two groups (χ² = 0.221, df = 1, p = 0.638).

From 97 laboratory-confirmed cases, a total of 103 parasites were identified. The 31-40-year age group had the most parasites isolated (24), followed by the 21-30-year age group (24 parasites isolated from 23 positive patients). There were a total of five cases of mixed infection with two or more parasites (Table 1). 

**Table 1:** Age-wise distribution of parasites isolated from cases (n= 103) Of 5,389 samples tested, 97 patients were confirmed positive for intestinal parasitic infections. Five patients had co-infections with two or more parasites; consequently, the total number of parasites isolated (n = 103) exceeds the number of positive cases (n = 97). *Column percentages are calculated out of 103 total parasites isolated. **Row percentages are calculated out of 103 total parasites isolated.

Parasite	Age in years							
	0-10	11-20	21-30	31-40	41-50	51-60	>60	Total*
*Giardia lamblia*	15 (14.70%)	5 (4.90%)	11 (10.78%)	8 (7.84%)	3 (2.94%)	2 (1.96%)	2 (1.96%)	46 (44.66%)
Hookworm	0	0	1 (0.98%)	5 (4.90%)	7 (6.80%)	3 (2.94%)	5 (4.90%)	21 (20.39%)
*Entamoeba histolytica/dispar/moshkovskii/bangladeshii* complex	0	1 (0.98%)	8 (7.84%)	5 (4.90%)	0	1 (0.98%)	2 (1.96%)	17 (16.50%)
*Blastocystis*	0	0	1 (0.98%)	2 (1.96%)	0	1 (0.98%)	1 (0.98%)	5 (4.85%)
*Cryptosporidium*	0	1 (0.98%)	0	1 (0.98%)	1 (0.98%)	0	0	3 (2.91%)
*H. nana*	0	1 (0.98%)	1 (0.98%)	0	1 (0.98%)	0	0	3 (2.91%)
*Cystoisospora belli*	0	0	1 (0.98%)	1 (0.98%)	0	0	0	2 (1.96%)
*Ascaris lumbricoides*	0	0	1 (0.98%)	1 (0.98%)	0	0	0	2 (1.94%)
*Strongyloides stercoralis*	0	0	0	0	0	1 (0.98%)	1 (0.98%)	2 (1.94%)
*Iodamoeba*	0	0	0	1 (0.98%)	0	0	0	1 (0.97%)
*Dipyllidium caninum*	0	0	0	0	1 (0.98%)	0	0	1 (0.97%)
Total**	15 (14.56%)	8 (7.77%)	24 (23.30%)	24 (23.30%)	13 (12.62%)	8 (7.77%)	11 (10.68%)	103

The study showed a higher positivity of protozoan infections (74/103; 71.84%) than helminthic infections (29/103; 28.16%). *Giardia lamblia *(46/103; 44.66%), hookworm (21/103; 20.39%), and the *Entamoeba histolytica/dispar/moshkovskii/bangladeshii* complex (17/103; 16.50%) were the three most frequently isolated parasites. *Dipylidium caninum *and *Iodamoeba* were the least commonly isolated parasites (1/103; 0.97% each), followed by *Cystoisospora belli*, *Ascaris lumbricoides*, and *Strongyloides stercoralis* (2/103; 1.94% each) (Table 2).

A combination of *E. histolytica *and *Blastocystis* sp. was the most commonly isolated combination (three of five mixed infections), followed by hookworm with *Giardia *and *E. histolytica *and *Iodamoeba *with *Cryptosporidium* spp. (one of five mixed infections each) (Table 2). 

**Table 2:** Pattern of intestinal parasites among the cases

Parasite detected	Number of cases
Single infection (n = 92)	
*Giardia lamblia*	45
Hookworm	20
*E. histolytica*	13
*Blastocystis*	2
*Cryptosporidium*	2
*H. nana*	3
*Cystoisospora belli*	2
*Ascaris lumbricoides*	2
*Strongyloides stercoralis*	2
*Dipyllidium caninum*	1
Multiple infection (n = 5)	
*E. histolytica* and *Blastocystis hominis*	3
Hookworm and *Giardia lamblia*	1
*E. histolytica*, *Iodamoeba*, and *Cryptosporidium *spp.	1

This study showed that the positivity rate among suspected individuals was higher before the COVID-19 pandemic (2.78%) than during the COVID-19 pandemic (1.48%). During COVID-19, the maximum positivity rate (1.67%) was recorded after ICMR removed ivermectin from the treatment regimen [7], compared with when it was included (1.13%) (Table 3). During the subsequent post-ivermectin-policy period, the positivity rate was 0.14% (95% CI: 0.03%-0.81%; based on a single positive case and therefore statistically unstable, not permitting clinical inference) from September to December 2021, followed by an increase to 2.24% (95% CI: 1.66%-3.02%) in 2022. 

**Table 3:** Year-wise distribution of study population and laboratory-confirmed cases of intestinal parasitic infections (N = 5,389 total samples; n = 97 positive patients) Sub-period positivity rate: positivity for each individual year or sub-year segment. Phase positivity rate: combined positivity for all rows within the same study phase. Overall COVID-era positivity rate: combined rate across the entire pandemic period (January 2020 to December 2022; 60/4,059 = 1.48%). NA: not applicable.

Period	Year	Months	Male	Female	Total N	Positive n	Sub-period positivity rate (%)	Phase positivity rate (%)	Overall COVID-era positivity rate (%)
Pre-COVID	2019	Jan–Dec	860	470	1,330	37	2.78	2.78	NA
Early COVID/Ivermectin-included	2020	Jan–Dec	361	190	551	9	1.63	1.14	1.48
Early COVID/Ivermectin-included	2021	Jan–Aug	591	351	942	8	0.85	1.14	1.48
Post-ivermectin	2021	Sep–Dec	447	245	692	1	0.14	1.68	1.48
Post-ivermectin	2022	Jan–Dec	1,146	728	1,874	42	2.24	1.68	1.48

The positivity rate of intestinal parasitosis differed significantly across the three study periods (p = 0.004). Post hoc pairwise analysis with Bonferroni correction (adjusted α = 0.017) demonstrated a statistically significant difference only between the pre-COVID period and the early COVID-19 pandemic period (p = 0.001; PR = 2.44, 95% CI: 1.38-4.32). The comparison between the pre-COVID and post-ivermectin periods was not significant after correction (p = 0.021; PR = 1.66, 95% CI: 1.08-2.56). The two pandemic-era periods showed no significant difference in positivity rates (p = 0.172; PR = 0.68, 95% CI: 0.39-1.19) (Tables 4, 5).

**Table 4:** Overall comparison of IPI positivity rates across the three study periods (α = 0.05) IPI: intestinal parasitic infection.

Timeline	Positive, n (%)	Negative, n(%)	Total tested, N	c² statistic	p-value
Pre-COVID	37 (2.78)	1293 (97.22)	1330	11.174 (df = 2; p = 0.004)	0.004
COVID Pandemic - Ivermectin included in treatment regimen	17 (1.14)	1476 (98.86)	1493		
COVID Pandemic - Post-ivermectin	43 (1.68)	2523 (98.32)	2566		

**Table 5:** Post-hoc pairwise comparisons of IPI positivity rates between study periods (Bonferroni-adjusted α = 0.017) *Statistically significant after Bonferroni correction. PRs were calculated with the earlier period as the reference (pre-COVID for pairs 1 and 3; early COVID for pair 2). All CIs are 95%. PR: prevalence ratio; OR: odds ratio; CI: confidence interval.

Timeline	Positive, n (%)	Negative, n( %)	c² statistic (degrees of freedom), p-value	PR (95% CI)	OR (95% CI)
First pair					
Pre-COVID	37 (2.78)	1293 (97.22)	10.123 (df = 1; p = 0.001*)	2.44 (1.38–4.32)	2.48 (1.39–4.43)
COVID Pandemic - Ivermectin included in treatment regimen	17 (1.14)	1476 (98.86)			
Second pair					
COVID Pandemic - Ivermectin included in treatment regimen	17 (1.14)	1476 (98.86)	1.869 (df = 1; p = 0.172)	0.68 (0.39–1.19)	0.68 (0.38–1.19)
COVID Pandemic - Post-ivermectin	43 (1.68)	2523 (98.32)			
Third pair					
Pre-COVID	37 (2.78)	1293 (97.22)	5.3296 (df = 1; p = 0.021)	1.66 (1.08–2.56)	1.68 (1.08–2.62)
COVID Pandemic - Post-ivermectin	43 (1.68)	2523 (98.32)			

While chi-square analysis with Bonferroni correction provides a conservative approach to multiple comparisons, it does not account for unequal period duration, variable testing volumes, seasonality, or temporal autocorrelation. We therefore report effect sizes as PRs and ORs with 95% CIs as supplementary measures of association magnitude. Retrospective laboratory records did not provide monthly denominator data in a format suitable for segmented binomial or Poisson regression modelling, which would have been the preferred approach for evaluating temporal level and trend changes. 

## Discussion

This study was conducted in the Parasitology Department of AIIMS, Rishikesh, presenting a four-year epidemiological analysis of intestinal parasitic infections among clinically suspected patients between January 2019 and December 2022. The study period includes both the pre-pandemic baseline and the COVID-19 pandemic, enabling an examination of temporal trends in laboratory positivity rates across three defined periods, while accounting for the confounding influence of reduced healthcare utilisation on observed rates and the consequent limitations on conclusions regarding ivermectin's antiparasitic activity.

The overall laboratory positivity rate for intestinal parasitic infections across the study period was 1.80%. Given the low absolute number of positive cases, a conservative statistical approach was adopted to minimise the risk of false-positive findings across multiple comparisons. Chi-square analysis with Bonferroni correction (adjusted α = 0.017) revealed a statistically significant decline in positivity rates between the pre-COVID period and the early COVID-19 period, the latter coinciding with the inclusion of ivermectin in the national treatment protocol (p = 0.001). The difference between the pre-COVID and post-ivermectin periods, while nominally significant, did not remain significant after correction (p = 0.021). No significant difference in positivity rates was observed between the two pandemic-era periods (p = 0.172).

The overall positivity rate among clinically suspected patients during these years was 1.80%, with a higher rate observed in the pre-COVID period (2.78%) than during the COVID-19 period (1.48%). This decline aligns with findings from studies conducted in Iran, Saudi Arabia, and Turkey [8]. According to an Iranian study, health education and personal hygiene could be the reason for the reduction in parasitic infections during the COVID-19 period [9]. According to a Saudi Arabian study, the government's pandemic quarantine policies may have contributed to the decline in infections [10]. A study conducted in Turkey stated that quarantine, personal hygiene, and public awareness can be effective in reducing parasitic infections during the COVID-19 period [11]. However, a contrasting study from Turkey reported a rise in the intestinal parasite detection rate during the pandemic [12], underscoring the regional variability in transmission dynamics.

The current study showed a higher positivity rate of protozoan infections (74 (72.54%)) than helminthic infections (29 (28.16%)). *Giardia lamblia *(46 (44.66%)), hookworm (21 (20.39%)), and the *Entamoeba histolytica/dispar/moshkovskii/bangladeshii* complex (17 (16.50%)) were the three most frequently isolated parasites. These results are consistent with studies conducted previously in various parts of India and Iran [9,13-15]. It is important to note that identification of the *Entamoeba histolytica/dispar/moshkovskii/bangladeshii* complex was based on conventional microscopy, and differentiation between pathogenic and nonpathogenic species was not possible in the absence of molecular or antigen-based assays. The detection of Dipylidium caninum in one adult patient represented an incidental but noteworthy finding. While human infection with this zoonotic cestode is uncommon [16], the route of acquisition in this case could not be ascertained and warrants further epidemiological inquiry.

Within-group positivity rates were highest among individuals in the 41-50-year age group (3.00%), followed by those in the 11-20-year and 0-10-year age groups. Although the 21-30-year age group accounted for the highest number of positive cases in the study, its within-group positivity rate was the lowest, reflecting its large representation in the tested population. Overall, most infections were detected in adults aged 21-40 years, possibly due to greater environmental, behavioural, and occupational exposure associated with this economically active age group. This age distribution is broadly consistent with findings from studies conducted in India [17] and Iran [9]. In contrast, another study conducted in India showed a high prevalence of intestinal parasites among individuals aged 16-30 years [15]. This difference may be due to variations in subject selection criteria and the geographical areas in which the studies were conducted.

Male patients accounted for nearly two-thirds (65.98%) of the positive cases, a trend commonly reported in parasitology studies and similar to findings reported by Meena et al. [15], Khanal et al. [18], Daryani et al. [19], and Teimouri et al. [9]. However, the lack of clearly defined criteria for “clinically suspected” cases is one of the limitations of this study. Although selection was based on clinician requests, the lack of written standardised symptom-based inclusion criteria may have affected the consistency of case identification.

The observed association with ivermectin use should be interpreted with caution. This study showed that positivity rates declined during the period when ivermectin was included in the national COVID-19 treatment protocol (1.13%) and increased after its withdrawal (1.67%). However, individual patient data on ivermectin use were unavailable, and the study periods were defined according to national treatment guidelines rather than confirmed drug administration. Consequently, the observed changes in positivity rates cannot be directly attributed to ivermectin. Other factors, including reduced healthcare utilisation, changes in health-seeking behaviour, public health measures, and evolving pandemic conditions, may also have influenced these trends.

The positivity rate of 0.14% (1/692; 95% CI: 0.03%-0.81%) in September-December 2021 rests on a single confirmed case. The extremely wide confidence interval renders this estimate statistically uninformative, and no inference regarding a residual antiparasitic effect of ivermectin should be drawn from this observation.

Although changes in positivity rates coincided temporally with periods of ivermectin use and withdrawal, the retrospective design and absence of individual-level treatment data preclude causal inference. Marked variation in sample volume across the study periods, along with changes in healthcare utilisation, public health restrictions, and health-seeking behaviour during the pandemic, may have contributed to the observed trends. Additionally, the use of conventional microscopy without concentration, antigen detection, or molecular methods may have reduced diagnostic sensitivity and underestimated parasite positivity. Therefore, the findings should be interpreted as temporal associations rather than evidence of a direct antiparasitic effect of ivermectin. The observed resurgence in positivity following the withdrawal period warrants continued surveillance of parasitic infections in the post-COVID era. The single-centre design and lack of individual-level exposure data may limit the generalizability of the findings.

Limitations

This study has several limitations that must be considered when interpreting the findings. First, the retrospective design and absence of standardised symptom-based referral criteria introduce selection bias; the composition of the clinically suspected population likely varied across study periods due to changing pandemic conditions, healthcare prioritisation, and patient-seeking behaviour. Second, individual-level ivermectin exposure could not be confirmed from laboratory records; the study periods are policy-defined and do not reflect verified drug administration. Third, the diagnostic methodology relied on conventional microscopy without concentration techniques, antigen-based detection, or molecular confirmation, likely underestimating the true burden of infection, particularly for *Giardia lamblia*, *Cryptosporidium *spp., and *Entamoeba* species. Fourth, modified Ziehl-Neelsen staining for coccidian parasites was performed on a reflexive rather than universal basis, introducing potential under-ascertainment of *Cryptosporidium* spp., *Cyclospora cayetanensis*, and *Cystoisospora belli*. Fifth, morphological identification of the *Entamoeba histolytica/dispar/moshkovskii/bangladeshii* complex did not permit differentiation between pathogenic and nonpathogenic species. Sixth, unmeasured environmental and behavioural confounders, including sanitation access, dietary practices, occupational exposure, and household crowding, were not captured in the retrospective laboratory dataset and may have independently influenced infection trends. Seventh, the low absolute number of positive cases (n = 97), including only one positive patient in September-December 2021, renders subgroup estimates statistically unstable; confidence intervals are wide, and caution is warranted in period-specific interpretations. Eighth, monthly denominator data were unavailable, precluding segmented regression analysis to evaluate temporal trends while adjusting for testing volume. Finally, the single-centre tertiary referral design limits the generalizability of these findings to community settings.

## Conclusions

This study documented a significant decline in the laboratory positivity rate for intestinal parasitic infections among clinically suspected patients at a tertiary referral centre during the early COVID-19 period, compared with the pre-pandemic baseline (PR = 2.44, 95% CI: 1.38-4.32). This decline most plausibly reflects reduced healthcare-seeking behaviour, altered patient referral patterns, and changes in the clinical prioritisation of stool examination during the pandemic, rather than a true community-level reduction in parasite burden. The lower positivity observed during the ivermectin-included period represents a temporal association only; the absence of individual-level treatment data, non-standardised referral criteria, and multiple uncontrolled confounders preclude causal inference, and this association must therefore be considered hypothesis-generating. The resurgence in positivity following the post-ivermectin period warrants continued epidemiological surveillance of intestinal parasitic infections as routine healthcare utilisation recovers in the post-pandemic era. Larger, multicentre, prospective studies with verified individual-level ivermectin exposure data, standardised inclusion criteria, and more sensitive diagnostic methods are needed to determine whether any direct association exists between ivermectin use and the incidence of intestinal parasitic infections.
